# Childhood Maltreatment and Depression in Adulthood in Chinese Female College Students: The Mediating Effect of Coping Style

**DOI:** 10.3389/fpsyt.2020.581564

**Published:** 2020-11-05

**Authors:** Zheng Zheng, Wenyue Han, Yuan Zhou, Ning Zhang

**Affiliations:** ^1^School of Medicine & Holistic Integrative Medicine, Nanjing University of Chinese Medicine, Nanjing, China; ^2^Nanjing Brain Hospital Affiliated to Nanjing Medical University, Nanjing, China

**Keywords:** childhood maltreatment, depression, coping style, mediating effect, psychological intervention

## Abstract

Depression is the most common psychological disorder of female, with high disability rate and remarkable mortality rate. There is a lack of knowledge about childhood experience, coping style, and adult depression. The aim of the present research was to enrich this knowledge by investigating the mediating effect of coping style between childhood maltreatment and depression in adulthood in Chinese female college students. Self-report questionnaires assessing childhood maltreatment, depression, and coping style were completed in 738 participants. The results illustrated that childhood maltreatment was positively related to depression in adulthood while coping style was negatively related to depression. In addition, childhood maltreatment could influence adult depression through the mediating role of coping style. These findings indicate that childhood maltreatment and negative coping style are associated with depression in adulthood. Psychological intervention strategies for coping style could provide effective treatment direction for depression caused by childhood maltreatment.

## Introduction

Depression is the leading cause of disability among people across the globe. It was estimated that the proportion of people suffering from depression in 2015 was 4.4%, and women (5.1%) were more common than men (3.6%) (1). Depression is common among the youth, and often indicates chronic and recurrent diseases in adulthood. A systematic review of medical students showed that the prevalence of depression or depressive symptoms was 27.2%, and the prevalence of suicidal ideation was 11.1% (2). Many psychosocial factors may affect depression, including cognitive impairment, stressor in life and circumstance, parental depression, interpersonal distress, and female gender (3). Childhood maltreatment, as one of the stressors in life, has been considered as a factor leading to depression (4). Therefore, the understanding of depression should not only consider its physiological susceptibility, but also focus on the individual's childhood experience and other psychosocial factors.

Childhood maltreatment (CM) includes many forms, involving emotional, physical and sexual abuse, as well as emotional and physical neglect (5). CM has been recognized as a crucial risk factor for depression. High levels of emotional and/or sexual abuse were associated with more significant severity of depression, and more markedly associated with depression than physical abuse (6). In other words, although each type of CM was positively correlated with the severity of depression, emotional abuse and emotional neglect were the strongest (7). The probability of depression in adulthood with CM experience was 2.66–3.73 times higher than that of normal people. The early onset age was more likely to develop into chronic or refractory depression (8). Although CM increases the risk of depression, not all abused children become depressed. Some modifiable factors increase vulnerability to, or act as a buffer against, depression. Coping style had a significant mediating effect between stressor and psychological distress (9). Maladjusted coping was the main predictor of depression and reduction of maladjusted coping behavior might have the most positive effect on the relief of depression (10).

To sum up, depression is a high incidence of psychological disorders, seriously endangering the health of female youth, even life-threatening. There are many causes of depression and CM is a crucial one. Many empirical studies have revealed the link between CM and depression. It is less clear what coping strategies might be used to assist individuals in decreasing depression cause by CM. In this research, we hypothesize that CM is not only an important predictor of adult depression, but also might be used to directly predict the severity of depression. And through the mediating effect of coping style, the severity of depression is indirectly affected.

## Methods

### Participants and Procedure

Seven hundred forty-five female college students were selected by cluster sampling. Seven hundred thirty-eight (99.1%) consented and took part in the current study, average age 19.4 years (SD = 1.1, range = 17–23). Two hundred eighty-four (38.5%) came from rural area and 454 (61.5%) from urban area. One hundred forty-six (19.8%) majored in science and engineering, 296 (40.1%) in medicine, 85 (11.5%) in art, and 211 (28.6%) in liberal arts.

The assessment was conducted in class under the supervision of research team members. The survey lasted 20 min. The study was approved by the Human Research Ethics Committee of NJUCM. Approval was also granted by each college. Research information was provided directly to participants and informed consent and consent were obtained.

### Measures

The Hospital Anxiety and Depression Scale [HADS; (11)] is a self-evaluation scale which examines anxiety and depression symptoms, respectively. The total score of depression or anxiety can be regarded as the severity of symptoms. HADS had good reliability and validity (12). In the current study, internal consistency α = 0.81.

The Simple Coping Style Questionnaire [SCSQ; (13)] contains 20 items assessing coping style which effectively reflect individual's coping style in the context of Chinese culture. Items 1–12 belong to positive coping and 13–20 belong to negative coping. The score of each item is from 0 (never) to 3 (always). If the average difference between positive coping and negative coping is >0, it is positive coping and <0 is negative coping. The internal consistency coefficient of the scale was 0.90 and the test-retest reliability was 0.89 (13). In the present study, total questionnaire internal consistency α = 0.75.

Personal Report of Childhood Abuse [PRCA; (14)] is compiled in the context of Chinese culture. It contains 20 items and measures the frequency and severity of abuse in childhood. The scale includes four sub-scales of physical abuse (PA), emotional abuse (EA), sexual abuse (SA), and neglect. Higher score indicates more severe CM. The Cronbach's α coefficient of the total scale and the four sub-scales of PRCA ranged from 0.604 to 0.839 in college students (15). In the current study, internal consistency α = 0.88.

### Statistical Analysis

All analyses were performed using SPSS22.0. All statistical tests were two-sided and the significance level was set at *p* < 0.05. Partial correlation was used to examine correlations among depression, CM and coping style. Because the scores of PRCA and subscales did not conform to normal distribution, Kruskal-Wallis method was used for nonparametric test to compare the differences in different depression status. The bootstrap method of Preacher and Hayes (16) and Hayes (17) was used to test the mediating effect.

## Results

### Correlations Among Childhood Maltreatment, Coping Style, and Adult Depression

The correlations among CM, adult depression and coping style are shown in Table 1. As revealed in the table, CM was positively related to adult depression, whereas coping style was negatively related to CM and adult depression.

**Table 1:** Partial correlation of various variables after controlling potential age effect.

	**1**	**2**	**3**	**4**	**5**	**6**	**7**
1. PRCA	1						
2. PA	0.816^***^	1					
3. EA	0.952^***^	0.723^***^	1				
4. SA	0.244^***^	0.224^***^	0.154^***^	1			
5. Neglect	0.751^***^	0.434^***^	0.588^***^	0.209^***^	1		
6. Coping style	−0.138^***^	−0.102^***^	−0.119^***^	−0.089^*^	−0.125^***^	1	
7. Adult depression	0.191^***^	0.119^***^	0.171^***^	0.106^***^	0.195^***^	−0.437^***^	1

*PRCA, personal report of childhood abuse; PA, physical abuse; EA, emotional abuse; SA, sex sbuse*.

* *p < 0.05 (two-tailed)*,

*** *p < 0.001 (two-tailed)*.

### The Relationship Between Childhood Maltreatment and Depression in Adulthood

According to the score of HADS, the participants were divided into three groups: asymptomatic (79.7%), suspicious depression (14.6%), and symptomatic depression (5.7%). The relationship between adult depression and CM is shown in Table 2. As revealed in the table, there were obvious differences in PRCA [χ^2^_(2, 735)_ = 22.06, *p* < 0.001], as well as emotional abuse [χ^2^_(2, 735)_ = 20.56, *p* < 0.001], sexual abuse [χ^2^_(2, 735)_ = 8.37, *p* < 0.05], and neglect [χ^2^_(2, 735)_ = 19.05, *p* < 0.001] among different depression status.

**Table 2:** Nonparametric test of childhood maltreatment at different depression status.

**Group**	**①**	**②**	**③**	***χ*^2^**
PRCA	1.99 ± 4.23	3.79 ± 5.83	4.64 ± 5.79	22.06^***^
PA	0.43 ± 1.14	0.59 ± 1.22	0.86 ± 1.64	3.61
EA	1.04 ± 2.37	1.94 ± 3.39	2.60 ± 3.47	20.56^***^
SA	0.01 ± 0.12	0.06 ± 0.32	0.07 ± 0.34	8.37^*^
Neglect	0.49 ± 1.15	1.13 ± 1.87	1.02 ± 1.60	19.05^***^

*PRCA, personal report of childhood abuse; PA, physical abuse; EA, emotional abuse; SA, sex abuse;* ①, *asymptomatic;* ②, *suspicious depression;* ③, *symptomatic depression*.

* *p < 0.05 (two-tailed)*,

*** *p < 0.001 (two-tailed)*.

### The Mediating Effect of Coping Style on the Relationship Between Childhood Maltreatment and Depression in Adulthood

**Figure 1:**
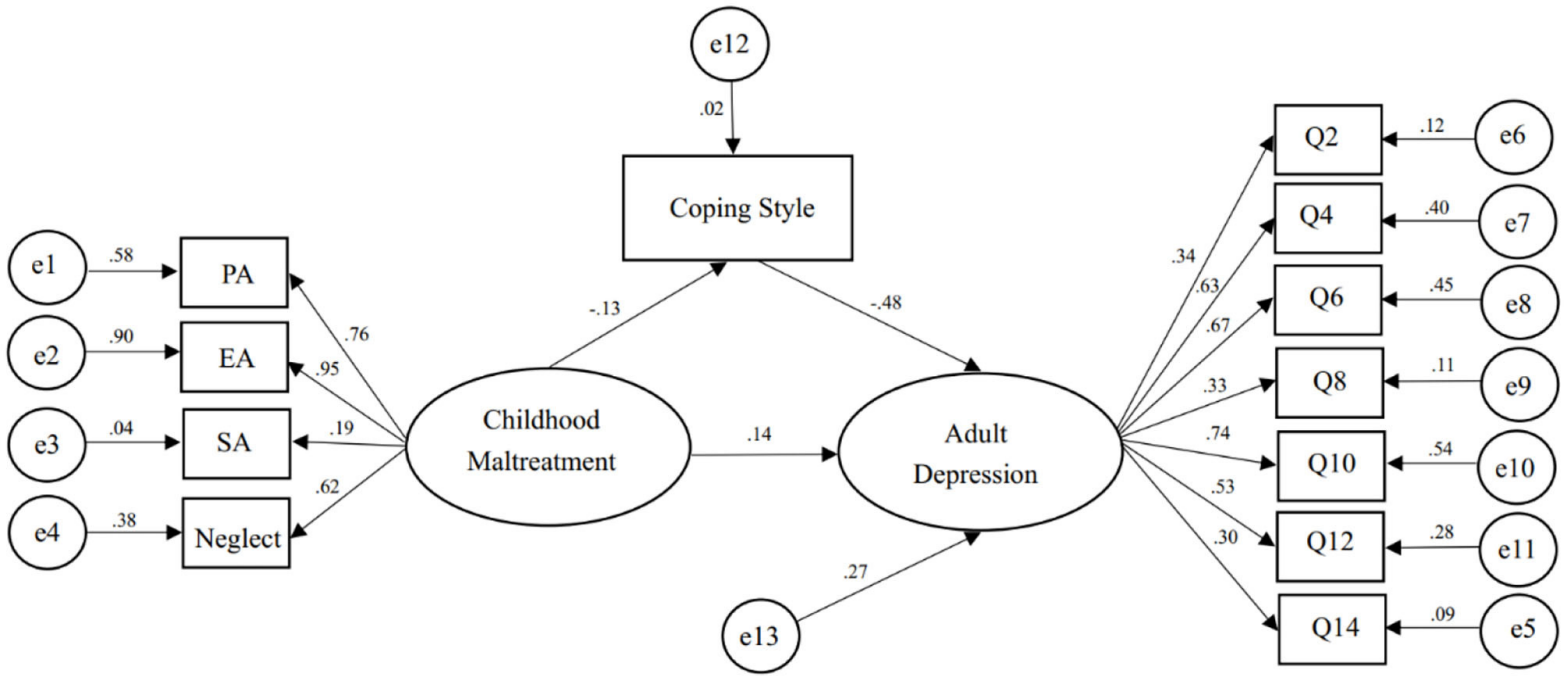
The path model of childhood maltreatment, coping style, and adult depression.

Taking CM as latent variable, the path model between CM, coping style and adult depression was constructed to verify the mediating effect of coping style (Figure 1). The fitting index of the model basically conformed to the standard of commonly used fitting statistics (Table 3). The standardized 95% CI of total effect of CM on depression was (0.109, 0.321), direct effect was (0.054, 0.244), indirect effects was (0.027, 0.107), excluding 0. Therefore, the overall effect, direct and indirect effects were significant. In the model, the overall effect of CM on adult depression was c = 0.207, a = −0.133, b = −0.476, c' = 0144, all of which reached a significant level. This presented that CM could not only directly predict adult depression, but also influence depression through the mediating effect of coping style. The direct effect (0.144) and mediating effect (0.063) accounted for 69.6 and 30.4% of the total effect (0.207), respectively (Table 4).

**Table 3:** Fitting index of intermediary model.

**χ^2^**	**df**	**χ^2^/df**	**RMSEA**	**GFI**	**NFI**	**IFI**	**CFI**
151.329	52	2.91	0.051	0.967	0.924	0.949	0.949

*df, degree of freedom; RMSEA, root mean square error of approximation; GFI, goodness of fit index; NFI, normed fit index; IFI, incremental fit index; CFI, comparative fit index*.

**Table 4:** Analysis of total effect, direct effect and indirect effect.

	**Effect value**	**Boot SE**	**Boot CI lower**	**Boot CI upper**	**Relative effect value**
Total effect	0.207	0.054	0.109	0.321	
Direct effect	0.144	0.041	0.054	0.244	69.6%
Indirect effect	0.063	0.020	0.027	0.107	30.4%

*Boot SE, Boot CI Lower, and Boot CI Upper refer to the standard error of indirect effect estimated by percentile bootstrap method corrected by deviation, the lower limit and upper limit of 95% confidence interval, respectively*.

Furthermore, four subscales of PRCA were used as independent variables and adult depression as dependent variable to test the mediating effects of coping style. The results showed that physical abuse (β = 0.12, *t* = 3.25, *p* < 0.01), emotional abuse (β = 0.17, *t* = 4.72, *p* < 0.01), sexual abuse (β = 0.11, *t* = 2.88, *p* < 0.01) and neglect (β = 0.20, *t* = 5.41, *p* < 0.001) had significant positive effects on adult depression. After adding coping styles, physical abuse (β = 0.08, *t* = 2.26, *p* < 0.01), emotional abuse (β = 0.12, *t* = 3.66, *p* < 0.001), sexual abuse (β = 0.07, *t* = 2.05, *p* < 0.05), and neglect (β = 0.14, *t* = 4.29, *p* < 0.001) still had significant effects on adult depression. The regression equations of the mediating effect of coping style on the four subscales of PRCA and adult depression is shown in Table 5.

**Table 5:** Regression equations of the mediating effect of coping style.

**Variable**	**Path**	**Regression equation**
PA, CS, AD	PA → CS	M = −0.10X
	PA, CS → AD	Y = 0.08X−0.43M
	PA → AD	Y = 0.12X
EA, CS, AD	EA → CS	M = −0.12X
	EA, CS → AD	M = −0.12X
	EA → AD	Y = 0.12X−0.42M
SA, CS, AD	SA → CS	M = −0.09X
	SA, CS → AD	Y = 0.07X−0.43M
	SA → AD	Y = 0.11X
Neglect, CS, AD	Neglect → CS	M = −0.13X
	Neglect, CS → AD	Y = 0.14X−0.42M
	Neglect → AD	Y = 0.20X

*PA, physical abuse; EA, emotional abuse; SA, sex abuse; CS, coping style; AD, adult depression*.

## Discussion

The consequences of CM can be short-term or even continue to adulthood, so it is a serious social and public health problem (18). CM is closely related to depression in adulthood. Therefore, it is important to understand the mechanism of their interaction for the provision of prevention and intervention services, which can help individuals with CM experience to better deal with emotional problems. CM is an important risk factor for health problems in adulthood, stress and coping strategies may affect this relationship (19). This study aims to explore the mediating role of coping style between CM and adult depression. In this study, we illustrate that compared with physical abuse, emotional abuse shows significant differences at different depression status, which is consistent with previous research (6). More importantly, the results indicate that coping style plays an exact mediating role between CM and adult depression. A study from Canada shows that positive coping strategies as a mediator can buffer the impact of childhood abuse on adult psychological distress, which is consistent with the results of this study, suggesting that the mediating role of coping styles between CM and adult depression may be cross-cultural (20). The findings of this study support the view that individuals who encounter CM will increase the severity of depression if they adopt negative coping style, while positive coping style can reduce the negative effects of CM to a certain extent. In addition, female youth with high degree of CM use more negative coping strategies and less adaptive coping skills, which affects their psychological health.

This study provides a valuable direction for the effective intervention of depression by investigating the mediating role of coping style on CM and adult depression. Coping is an individual's conscious, purposeful and flexible adjustment behavior to the change of real environment. Individuals can evaluate and regulate the physical and emotional responses related to stress events by coping. Although people may suffer different kinds of stress events, their coping styles are often stable and consistent. Some tend to adopt positive strategies, such as seeking support and changing the belief system, while others tend to adopt negative strategies, such as avoidance and emotional venting. Previous studies also found that inappropriate coping strategies such as self-blame, denial and abandonment were the main predictors of depression, anxiety, and stress (10). This study illustrated that coping style can be used as an intermediary variable to regulate the effect of CM on adult depression. For any person, childhood experience is an established fact that has happened and can't be changed. However, coping style can be changed through purposeful training and intervention as to actively adjust the individual's mental and physical state.

In this study, we tested the hypothesis that CM not only positively predicted adult depression, but also indirectly affected adult depression through coping style. The contribution of this study is to verify the mediating effect of coping style on CM and adult depression, which can provide guidance for psychological intervention of depression. The limitation of this study is that how to adjust coping strategies to alleviate depression needs more empirical research. In addition, the subjects of this study are female college students, and the sampling scope will be further expanded in the future. Moreover, considering the differences of cultural factors in various countries on the specific manifestations of CM, we chose the questionnaire compiled by Chinese scholars. In future research, we might try to select tools with more items and details, such as Childhood Trauma Questionnaire (CTQ) to verify whether there would be more findings.

## Data Availability Statement

The original contributions generated for the study are included in the article/Supplementary Materials, further inquiries can be directed to the corresponding author/s.

## Ethics Statement

The studies involving human participants were reviewed and approved by the Human Research Ethics Committee of NJUCM. The patients/participants provided their written informed consent to participate in this study.

## Author Contributions

ZZ is responsible for design and writing. WH and YZ are responsible for questionnaire test and data analysis. NZ is for revising. All authors contributed to the article and approved the submitted version.

## Conflict of Interest

The authors declare that the research was conducted in the absence of any commercial or financial relationships that could be construed as a potential conflict of interest.

## References

[1] WHO Depression and Other Common Mental Disorders, Global Health Estimates. World Health Organization (2017). Available online at: http://apps.who.int/iris/bitstream/10665/254610/1/WHO-MSD-MER-2017.2-eng.pdf

[2] Rotenstein LS Ramos MA Torre M Segal JB Peluso MJ Guille C. Prevalence of depression, depressive symptoms, and suicidal ideation among medical students: a systematic review and meta-analysis. JAMA. (2016) 316:2214–36. 10.1001/jama.2016.17324 27923088 PMC5613659

[3] Hammen C. Risk factors for depression: an autobiographical review. Annu Rev Clin Psychol. (2018) 14:1–28. 10.1146/annurev-clinpsy-050817-084811 29328780

[4] Park C Rosenblat JD Brietzke E Pan Z Lee Y Cao B. Stress, epigenetics and depression: a systematic review. Neurosci Biobehav Rev. (2019) 102:139–52. 10.1016/j.neubiorev.2019.04.010 31005627

[5] Liu RT Scopelliti KM Pittman SK Zamora AS. Childhood maltreatment and non-suicidal self-injury: a systematic review and meta-analysis. Lancet Psychiatry. (2018) 5:51–64. 10.1016/S2215-0366(17)30469-8 29196062 PMC5743605

[6] Vallati M Cunningham S Mazurka R Stewart JG Larocque C Milev RV. Childhood maltreatment and the clinical characteristics of major depressive disorder in adolescence and adulthood. J Abnorm Psychol. (2020). 129:469–79. 10.1037/abn0000521 32237880

[7] Humphreys KL LeMoult J Wear JG Piersiak HA Lee A Gotlib IH. Child maltreatment and depression: a meta-analysis of studies using the Childhood Trauma Questionnaire. Child Abuse Negl. (2020) 102:104361. 10.1016/j.chiabu.2020.104361 32062423 PMC7081433

[8] Janna N Anne K Philipp D Ehring T. Childhood maltreatment and characteristics of adult depression: meta-analysis. Br J Psychiatry. (2017). 210:96–104. 10.1192/bjp.bp.115.180752 27908895

[9] Yan W Ping W. Perceived stress and psychological distress among Chinese physicians: the mediating role of coping style. Medicine. (2019) 98:e15950. 10.1097/MD.0000000000015950 31169719 PMC6571215

[10] Mahmoud JS Staten R Hall LA Lennie TA. The relationship among young adult college students' depression, anxiety, stress, demographics, life satisfaction, and coping styles. Issues Ment Health Nurs. (2012) 33:149–56. 10.3109/01612840.2011.632708 22364426

[11] Zigmond AS Snaith RP. The hospital anxiety and depression scale. Acta Psychiatr Scand. (1983) 67:361–70. 10.1111/j.1600-0447.1983.tb09716.x 6880820

[12] Olssøn I Mykletun A Dahl AA. The hospital anxiety and depression rating scale: a cross-sectional study of psychometrics and case finding abilities in general practice. BMC Psychiatry. (2005) 5:46. 10.1186/1471-244X-5-46 16351733 PMC1343544

[13] Yaning X. Preliminary study on the reliability and validity of the Simplified Coping Style Questionnaire. Chin J Clin Psychol. (1998) 6:114–5. 30518288

[14] Xianghua Z Jiao L Yongjie Y Xianyu W Yuxiang T Juan Q Personal report of childhood abuse reliability and validity in a community. Chin J Behav Med Sci. (2006) 15:1045–7.

[15] Xianghua Z Guangli L Juan Q Lin L Houfeng Z Yongjie Y Reliability and validity of the personal report of childhood abuse in college students. Chin J Health Psychol. (2011) 19:959–61. Available online at: https://en.cnki.com.cn/Article_en/CJFDTotal-JKXL201108032.htm

[16] Preacher KJ Hayes AF. SPSS and SAS procedures for estimating indirect effects in simple mediation models. Behav Res Methods Instrum Comput. (2004) 36:717–31. 10.3758/BF03206553 15641418

[17] Hayes AF Introduction to Mediation, Moderation, and Conditional Process Analysis: A Regression-Based Approach. New York, NY: The Guilford Press (2012).

[18] Arslan G. Psychological maltreatment, coping strategies, and mental health problems: a brief and effective measure of psychological maltreatment in adolescents. Child Abuse Negl. (2017) 68:96–106. 10.1016/j.chiabu.2017.03.023 28427000

[19] Hager AD Runtz MG. Physical and psychological maltreatment in childhood and later health problems in women: an exploratory investigation of the roles of perceived stress and coping strategies. Child Abuse Negl. (2012) 36:393–403. 10.1016/j.chiabu.2012.02.002 22609072

[20] Su Y D'Arcy C Meng X. Social support and positive coping skills as mediators buffering the impact of childhood maltreatment on psychological distress and positive mental health in adulthood: analysis of a national population-based sample. Am J Epidemiol. (2020) 189:394–402. 10.1093/aje/kwz275 31907548

